# Effects of anterior chamber depth and axial length on corneal endothelial cell density after phacoemulsification

**DOI:** 10.12669/pjms.35.1.92

**Published:** 2019

**Authors:** Muhammad Khalid, Sameer Shahid Ameen, Nauman Ayub, Muhammad Asim Mehboob

**Affiliations:** 1 Dr. Muhammad Khalid, MBBS, Department of Ophthalmology, PNS SHIFA, Karachi, Pakistan; 2 Dr. Sameer Shahid Ameen, FCPS, Department of Ophthalmology, PNS SHIFA, Karachi, Pakistan; 3 Dr. Nauman Ayub, FCPS, Department of Ophthalmology, PNS SHIFA, Karachi, Pakistan; 4 Dr. Muhammad Asim Mehboob, FCPS, Department of Ophthalmology, PNS SHIFA, Karachi, Pakistan

**Keywords:** Phacoemulsification, Corneal Endothelial Cell Density, Anterior Chamber Depth, Axial Length

## Abstract

**Objective:**

To evaluate mean decrease in Corneal Endothelial cell Density (CED) after phacoemulsification in patients with different Anterior Chamber Depths (ACDs) and Axial Lengths (ALs).

**Methods:**

This prospective stratified controlled study was conducted at PNS Shifa Hospital, Karachi. One hundred eyes of 90 patients, scheduled to undergo phacoemulsification surgery, were included. AL and ACD of each patient were calculated preoperatively using IOL Master. Cataracts were classified according toLens Opacities Classification System III (LOCS III) giving nuclear opalescence (NO) grades on slit lamp examination and only patients with grades NO2 and NO3 were included. Eyes were divided into two groups according to ACD and AL: Group-I: ACD 2.0mm – 3 mm and AL 22mm – 23.5mm; Group-II: ACD 3.1 mm -4.0 mm and AL 23.6mm – 25mm. CED measurements were done preoperatively and 2 month postoperatively using specular microscopy. The difference in CED change (Endothelial Cell Loss) between the two groups after surgery was analyzed using SPSS, v 22; IBM Corporation, Armonk, NY.

**Results:**

Differences in gender, laterality, age and preoperative CED between two groups were not significant. Difference in postoperative CED was also not significant, however difference in mean change and mean frequency change in CED between two groups was found to be statistically significant.

**Conclusion:**

ACD and AL affect the CED during phacoemulsification and Intraocular Lens(IOL) implantation and can be considered as risk factors of peroperative endothelial cell loss.

## INTRODUCTION

Corneal endothelial cells subserve a very important role of controlling corneal hydration by their pump mechanism, apart from other functions. Their loss below critical number (<500 cells/mm^2^) can lead to corneal edema, and decreased corneal transparency with subsequent decreased visual acuity.^1^,^2^ Nature has provided an adequate number of endothelial cells (Average 2500 cells/mm^2^ in adults) so that the integrity of cornea is ensured.^1^ The number of cells decrease with age at different rates as calculated in different studies;^3^,^4^,^5^ it is generally accepted to be about 0.6% per year. These cells typically can not replicate. When they are lost, the remnant endothelial cells compensate this by their migration, enlargement, and increasing heterogeneity.^6^ Such corneal decompensation is a rare but potentially vision-threatening complication of phacoemulsification surgery. Corneal endothelial cell density (CED) can be used as an indicator of the corneal trauma resulting from operation. A number of studies have stated that some preoperative and operative parameters can increase the risk of CED loss after phacoemulsification; those include Nucleus Opalescence (NO), ultrasound energy, phacoemulsification time, phacoemulsification technique, corneal tunnel length, irrigation solution turbulence, instrument-related trauma, Anterior Chamber Depth (ACD) and Axial Length (AL).^6^-^8^ Accordingly, the assessment of such risk factors is very important for the surgeons to be more cautious during surgery.

The importance of predetermined ACD and AL cannot be overlooked. Low ACD and AL indicate low working space for the phacoemulsification surgery, which involves use and maneuver of fine instruments. Therefore, the mechanical as well as thermal effects during surgery can damage corneal endothelium.

There are studies which have established the effect of ACD is not significant in endothelial cell loss after phacoemulsification surgery.^9^,^10^ Yet, no stratified controlled study has compared CED loss according to different ACDs and ALs. In our study we evaluated CED loss according to different ACDs and ALs in patients with specific cataract NO.

## METHODS

After approval by the hospital ethical review committee, this prospective stratified controlled study was conducted in Department of Ophthalmology, PNS Shifa hospital Karachi from Nov 2016 to Nov 2017. Initially 118 eyes of 102 patients scheduled to undergo phacoemulsification surgery with IOL Implantation were enrolled. Eight patients (14 eyes) were lost to follow up and four patients (4 eyes) developed postoperative complications. Therefore total 100 eyes of 90 patients were finally included in the study. Informed written consent was taken from all the patients prior to inclusion in the study. Patients fulfilling the following inclusion criteria were included and those meeting the excluding criteria were abstained.

### Inclusion Criteria

- Patients aged between 45-65 years
- Cataract density of NO2 and NO3
- Central Corneal Thickness (CCT) from 490µm to 550 µm
- Astigmatism of less than 2D

### Exclusion Criteria

- History of ocular surgery
- History of ocular diseases like glaucoma, uveitis etc
- History of ocular trauma
- Active Inflammation
- Keratoconus
- Corneal edema
- Cataract density NO1 and NO4
- CCT less than 490µm
- CED less than 2000 cells/mm^2^
- Intraoperative complications, such as posterior capsule rupture
- Postoperative complications
- Inability to maintain fixation during specular microscopy
- Prolonged phaco time (>45 sec)

Before surgery, all subjects underwent complete ophthalmic examination including visual acuity measurement and slit lamp examination. Further evaluation included automated refraction using Canon RK-F1 Full Auto Ref-Keratometer, three measurements of CCT and CED using Topcon SP 3000P Specular Microscope (Topcon Corporation, Tokyo, Japan) and measurement of ALs and ACDs using IOL Master®.

Eyes were divided into two groups according to ACD and AL: Group-I: ACD 2.0mm – 3.0mm and AL 22mm – 23.5mm; Group-II: ACD 3.1 mm -4.0 mm and AL 23.6mm – 25mm. All cataracts were graded according to Lens Opacities Classification System III (LOCS III) and only those with Nuclear Opalescence 2 and 3 (NO-2 and NO-3) were included.

After preoperative recordings, the subjects underwent successful phacoemulsification surgery and posterior chamber IOL implantation. Phacoemulsification with IOL implantation was performed in all patients by same surgeon using same phaco machine (Oertli, Faros, Switzerland) and operation room set up under local anaesthesia (peribulbar) with 2% lignocaine. In all cases combination of 1% sodium hyaluronate (Provisc, Alcon) and 2% hydroxypropyl methylcellulose (Viscot, Alcon) was used as viscoelstics. Phaco power and phaco time were recorded for each patient.

All patients were re-examined two month postoperatively. Again three readings of CED and CCT were obtained. All data was recorded using pre-devised proforma for record keeping. Statistical package for social sciences (SPSS 22.0) for windows was used for statistical analysis. Descriptive statistics i.e. mean ± standard deviation for quantitative values (age, CCT, ACD, AL, CED) and frequencies along with percentages for qualitative variables (gender, laterality) were used to describe the data. Shapiro Wilk’s test was used to check normality of data. Post normality testing, Chi square test was used to compare qualitative variables, and independent t test to compare quantitative variables between two groups. Moreover, Paired ‘t’ test was used to compare postoperative value from pre-operative value within each group. P-value < 0.05 was considered statistically significant.

## RESULTS

Mean age, gender distribution, laterality of eyes, AL, ACD, pre-operative CED, post-operative CED, mean change in CED and mean frequency change in CED of study population, and of both groups is given in Table-I. The difference in laterality, age and preoperative CED, and post-operative CED between two groups was not statistically significant. However, difference in mean change in CED and mean frequency change in CED between two groups was found to be statistically significant (p = <0.05). The comparison of pre-operative CED from post-operative CED within each group is given in Table-II. Difference of pre-operative CED from post-operative CED in both groups was statistically significant.

**Table-I:** Group wise demographic data (n=100).

Characteristic	Study Population (n=100)	Group-1 (n=50)	Group-2 (n=50)	p-Value (Between groups)
Age (Years) Mean ± SD	62.90± 7.17	62.68±7.03	63.12± 7.32	0.726**
Gender (Male/Female)	58 / 42	24 / 26	34 / 16	0.11*
Laterality (Right/Left)	60/40	34/16	26/24	0.05*
AL (mm) Mean ± SD	23.26±0.61	22.87±0.31	23.64±0.59	< 0.001**
ACD (mm)	3.06±0.43	2.73±0.23	3.3868±0.32	< 0.001**
Pre-Op CED (Cells/mm2) Mean ± SD	2768.38±327.03	2766.83±339.56	2769.932±315.84	0.963**
Post-Op CED (Cells/mm2) Mean ± SD	2555.3±345.71	2507.45±315.98	2603.148±368.19	0.168**
Mean Change in CED (Cells/mm2) Mean ± SD	213.08±199.44	259.38±116.78	166.7840±248.44	0.019**
Mean Frequency Change in CED (%)Mean ± SD	7.61±6.59	9.30±3.99	5.9197±8.07	0.010**

* Chi Square test,

** Independent t-test

**Table-II:** Pre-op and post-op comparison between two groups.

Variable	Group-1 (n=50)	Group-2 (n=50)
CED (Cells/mm^2^) mean ± SD		
Pre-operative	2766.83±339.56	2769.932±315.84
Post-operative – 2months	2507.45±315.98	2603.148±368.19
p-Value*	<0.001	<0.001

* Paired t-test.

## DISCUSSION

Corneal endothelial cells are key indicators of corneal integrity and function. They constantly dehydrate the cornea and also form the descemet membrane. Their significance in healthy as well as diseased has been emphasized greatly during last few decades after invent of reliable machines for analyzing endothelial cell morphology and function. A specular microscope is a reflected light microscope that works by projecting light onto the cornea and then imaging the reflected light from optical interface between corneal endothelium and aqueous humor. Those images are analyzed by the machine and give us different endothelial cell parameters like number, density, variation in size and hexagonality. In our study we have analyzed the endothelial cell number loss only, among other endothelial cell parameters.

Corneal endothelial cell loss (CED loss) leads to loss of hexagonality of surrounding cells intended to fill the space.^6^ With greater loss, the pump mechanism of remaining cells is insufficient to dehydrate cornea and edema ensures (compromised cornea); leading to decreased corneal transparency. This greatly affects the visual acuity of the patient, which eventually becomes a permanent loss.

CED loss during phacoemulsification is unavoidable. It is mainly due to very small confined space (Anterior Chamber) where surgery is performed. Corneal endothelium comes in direct contact with instruments multiple times during surgery making them vulnerable to damage by high ultrasound energy of phaco tip. Hence it is presumed that shallow anterior chamber is a risk factor for CED loss preoperatively and deeper anterior chamber is safer comparatively.

CED loss has been studied with respect to various modifiable and non modifiable factors. We have conducted our study with focus on anatomical non modifiable factor such as AL and ACD. We used IOL Master® for determination of AL and ACD. Other non modifiable risk factors for CED loss include diabetes mellitus, age and cataract density. The modifiable risk factors for CED loss include corneal incision size, phacoemulsification time, mean ultrasound power, irrigation solution turbulence, instrument-related trauma, type of viscoelastic substances being used, nuclear fragments and IOL contact during insertion and the type of implanted IOL.^6^-^8^

Cataract density is a non modifiable risk factor for CED loss. As can be expected, dense cataracts require more energy and time to break with greater manipulation within the limited chamber; making corneal endothelium vulnerable to damage. We selected cases with moderate cataract densities (NO-2 and NO-3) in our study to avoid major differences in surgical handling and techniques. Moreover, same phaco machine was used and same surgeon performed all surgeries with same surgical technique. This plays down chances of partiality in results.

A number of studies have precluded that AL and ACD influence the CED loss.^11^,^12^ Others have turned down any effect of these parameters on endothelial damage.^9^,^10^ Cho et al. measured the decrease in CED in different anterior chamber depth groups, with no significant difference of CED loss within these groups.^13^ In the study by Hwang BH et al., CED loss was higher in smaller ACD group as compared to large ACD groups, but the results were not statistically significant.^14^ In present study we divided the cases undergoing phacoemulsification into two groups according to ACD and AL: Group-I: ACD 2.0mm – 3.0mm and AL 22mm – 23.5mm; Group-II: ACD 3.1 mm -4.0 mm and AL 23.6mm – 25mm. We documented statistically significant effect of shallow anterior chamber and smaller AL on CED loss (p = <0.05). This is the first study in Pakistan that has been conducted on specific parameters and their influence on one parameter i.e. CED loss. We hope this study will help phaco surgeons in planning surgeries in eyes with shallow anterior chambers with greater care and better techniques. Moreover, this will open doors for research on better and safer techniques in shallow anterior chambers and small eyes.

### Author`s Contribution

**MK** conceived, designed, prepared & edited manuscript.

**SSA and NA** did editing & final approval of manuscript.

**MAM** did statistical analysis & review of manuscript.
